# The patient journey toward a diagnosis of hereditary transthyretin (ATTRv) amyloidosis

**DOI:** 10.1186/s13023-020-01623-1

**Published:** 2021-01-11

**Authors:** Montserrat Vera-Llonch, Sheila R. Reddy, Eunice Chang, Marian H. Tarbox, Michael Pollock

**Affiliations:** 1 Global Health Economics and Outcomes, 22 Boston Wharf Road, 9th Floor, Boston, MA 02210 USA; 2 Partnership for Health Analytic Research, LLC, 280 S. Beverly Dr., Ste 404, Beverly Hills, CA 90212 USA

**Keywords:** Amyloidosis, Healthcare utilization, Diagnosis, Patterns of care

## Abstract

**Background:**

Despite emerging treatments for hereditary transthyretin (ATTRv) amyloidosis, the disease is often misdiagnosed, with reported diagnostic delays of up to several years. Knowledge of the patient journey leading up to diagnosis may help to promote earlier intervention. The study’s objective was to examine patient clinical characteristics and healthcare utilization prior to ATTRv amyloidosis diagnosis.

**Methods:**

Patients ≥ 18 years and newly diagnosed with ATTRv amyloidosis identified in IBM® MarketScan® Commercial and Medicare Supplemental data using a claims-based algorithm as follows: diagnosis required ≥ 1 medical claim with relevant amyloidosis diagnosis code (ICD-10-CM: E85.0-.4, E85.89, E85.9; excludes light chain and wild type) during identification (ID) period (1/1/2016–12/31/2017), and ≥ 1 occurrence of qualifying criteria during 2011–2017: ≥ 15 days diflunisal use without > 30-day gap, liver transplant, or claim with specific codes E85.1 or E85.2. The index date was defined as the date of first claim with amyloidosis diagnosis code in ID period. Patients had continuous enrollment ≥ 5 years pre-index date (look-back period). Occurrence of selected comorbid conditions and symptoms and healthcare utilization (testing, emergency department visits and hospitalization) measured during the look-back period; demographics, physician specialty, and Charlson comorbidity index (CCI) measured 1 year pre-index. Patients with an ICD-9/10 amyloidosis code during the look-back period were excluded. An ATTRv-free reference cohort was created from a random sample of enrollees who lacked any diagnosis of amyloidosis and matched 3:1 to ATTRv patients on age, gender, and region to provide reference values; same index and enrollment requirement as match.

**Results:**

For the 141 qualifying patients with ATTRv and 423 matched controls, mean (standard deviation) age was 62.5 (14.2) years and 53.9% were female. Mean CCI for ATTRv cohort was 2.7 (3.0) versus 1.1 (1.9) among controls. Selected comorbidities, testing, visits, and hospitalization were common among patients with ATTRv during the look-back period with higher rates versus controls.

**Conclusions:**

Patients with ATTRv amyloidosis experience multiple neurological, cardiovascular, and other clinical manifestations, testing, and hospitalization prior to diagnosis. Occurrence of potential markers of illness is most common in the year before diagnosis.

## Background

Hereditary transthyretin (ATTRv) amyloidosis is a rare, progressive, multisystemic, and fatal form of amyloidosis caused by extracellular deposition of transthyretin amyloid fibrils primarily synthesized by the liver [1, 2]. The United States (US) prevalence of ATTRv amyloidosis with polyneuropathy has been reported to range from 2488 to 6400 patients [3, 4]. However, an estimated prevalence of 3–4% among African Americans has also been reported primarily presenting with signs and symptoms of cardiomyopathy [5, 6]; a recent long-term population-based study of Val122Ile carriers report clinically penetrant disease of approximately 20% suggesting about 25,000 affected individuals in the US [7]. Prevalence estimates are likely underestimated due to diagnostic uncertainty [1, 8, 9].

Symptoms of ATTRv amyloidosis can impact a variety of organ and body systems, including ocular, musculoskeletal, gastrointestinal (GI), and cardiac impairment. Variability in symptom manifestation, along with the lack of disease awareness amongst healthcare professionals, can lead to misdiagnoses. For example, polyneuropathy involvement may be mistakenly attributed to other neuropathic conditions, rather than ATTRv amyloidosis, leading to multiple misdiagnoses [1, 10, 11]. Reports of misdiagnoses range from 32 to 74% of patients with ATTRv amyloidosis, with 18% having received multiple misdiagnoses [12–16]. Time from symptom onset to ATTRv amyloidosis diagnosis is often 3 years or more [17–19]. Strategies for earlier identification include tissue biopsy and cardiac imaging; however, diagnostic delay is still common and can lead to poorer outcomes and greater disease burden [1, 10, 11, 17, 20, 21]. If untreated, death occurs typically 3–15 years after clinical presentation [1]. Early intervention is also key for effective treatment as the aim is to prevent additional amyloid deposition [10, 11, 22, 23]. Liver transplant was standard of care until recently, when TTR stabilizers and gene silencers entered the market [10, 22]. Several novel pharmacological options, including RNA interference therapy (e.g., patisiran), and antisense oligonucleotides (e.g., inotersen) have shown efficacy in treating the early stages of ATTRv amyloidosis with polyneuropathy [10, 11, 23].

While limited research exists about the patient journey prior to diagnosis for other forms of amyloidosis, such as light chain (AL) and wild-type amyloidosis [24–27], we identified no real-world studies in patients with ATTRv amyloidosis. The aim of this study was to examine the occurrence and timing of patients’ clinical presentation and healthcare utilization prior to diagnosis of ATTRv amyloidosis in the US.

## Methods

This study was a retrospective claims analysis of IBM® MarketScan® Commercial and Medicare Supplemental Databases.^1^ The MarketScan databases represent health services of more than 41.1 million employees, dependents, and retirees in the US with primary or Medicare supplemental coverage through privately insured fee-for-service, point-of-service, or capitated health plans. The databases include de-identified enrollment information and adjudicated insurance claims with information on healthcare utilization, including inpatient and outpatient services and prescription drug dispensing collected from employers and health plans who have agreed to be data contributors [28, 29]. Each individual in the database is assigned a unique enrollee identifier, created by encrypting information provided by data contributors. These databases are designed to address the requirements of the Health Insurance Portability and Accountability Act of 1996 (HIPAA); contain none of the data elements prohibited by HIPAA for such data sets; and have also gone through a third party statistical analysis to verify that they meet HIPAA requirements for fully de-identified data sets [29]. Thus, institutional review board approval was not required because subjects in this database cannot be identified. Meeting these conditions makes this research exempt from the requirements of 45 CFR 46.101 under the Department of Health and Human Services [30].

**Fig. 1:**
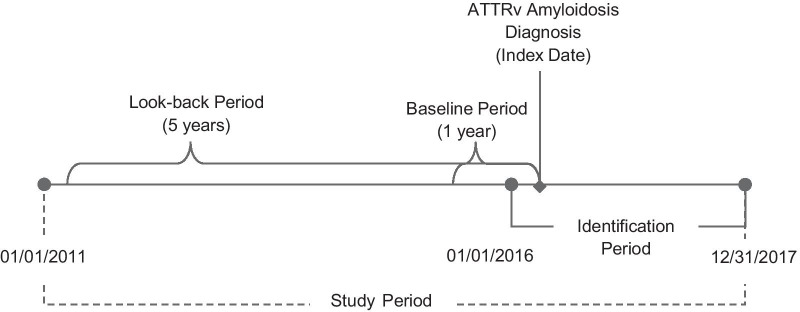
Study time frame. *ATTRv* hereditary transthyretin

Adult patients, at least 18 years old, who were newly diagnosed with ATTRv amyloidosis were identified by the presence of at least one medical claim (inpatient or outpatient) for amyloidosis, except light chain or wild type, in any diagnosis field (International Classification of Diseases, 10th Revision, Clinical Modification [ICD-10-CM]: E85.0, E85.1, E85.2, E85.3, E85.4, E85.89, E85.9) during the identification (ID) period (01/01/2016–12/31/2017); and either (1) use of diflunisal for at least 15 days or (2) liver transplant during the study period (01/01/2011–12/31/2017). The duration of diflunisal use was selected to eliminate use in the treatment of short-term pain. Patients with E85.1 or E85.2, the most specific codes for hereditary forms of amyloidosis, did not require an additional qualifier. Patients were excluded if not continuously enrolled in a health plan for at least 5 years prior to the index (look-back period). Additionally, to ensure patients were newly diagnosed, patients were excluded if they had an ICD-9-CM (i.e., 277.30, 277.31, and 277.39) or ICD-10-CM code for amyloidosis during the look-back period. The index date was the first diagnosis of amyloidosis in the ID period (Fig. 1, Table 1).

**Table 1:** Patient attrition

	Criteria	N
A	Adult patients with ≥ 1 medical claim with relevant amyloidosis diagnosis code (ICD-10-CM: E85.0-.4, E85.89, E85.9; excludes light chain and wild type) during identification period (01/01/2016–12/31/2017). The first diagnosis date is defined as index date	6115
B	Of A, with ≥ 1 occurrence of qualifying criteria during study period: ≥ 15 days diflunisal use with > 30-day gap OR liver transplant (patients with claim with specific codes E85.1 or E85.2 at any time did not require additional qualifier)	302^a^
C	Of B, who were newly diagnosed (i.e., had no ICD-9/10 amyloidosis code during look-back period) and had continuous enrollment during 5 years pre-index date (look-back period)	141

^a^279 patients with a claim with E85.1 or E85.2, 26 with ≥ 15 days diflunisal use, and 6 with liver transplant; some patients may have had multiple qualifiers

To serve as a reference group, an ATTRv amyloidosis-free control group was derived from a random sample of enrollees who lacked any diagnosis of amyloidosis during the study period and matched 3:1 to patients with ATTRv amyloidosis based on age, gender, and region. The amyloidosis-free cohort had the same index and health plan enrollment requirement as matched patients with ATTRv amyloidosis.

In each pre-index year during the 5-year look-back period, the occurrence of selected comorbid conditions and symptoms, which can also manifest as complications of ATTRv amyloidosis, and healthcare utilization (i.e., diagnostic testing [blood/urine testing, pyrophosphate imaging, cardiac magnetic resonance imaging, echocardiogram, and tissue biopsy or genetic testing], emergency department [ED] visits and hospitalizations) were identified using ICD diagnosis and procedure codes, current procedural (CPT) codes, and medical claims. Baseline (1 year prior to the index date) variables were also identified for patient demographic and clinical characteristics, such as age, gender, region, insurance, Charlson comorbidity index (CCI) as a proxy measure for underlying health status and risk of mortality [31, 32], and physician specialty. The CCI is a method of categorizing comorbidities of patients based on the ICD diagnosis codes found in administrative data [31, 32].

Descriptive statistics were generated for pre-index measures (first or any occurrence) during each year of the look-back period. Means and standard deviations (SD) were used to summarize continuous variables; and relative frequencies and percentages were used to summarize categorical variables. Cumulative probability curves were used to summarize the successive probability of occurrence of selected comorbidities and testing during the look-back period. To compare patients with ATTRv amyloidosis to patients free of the disease, *t* test and Chi-square test were conducted for continuous variables and categorical variables, respectively. All data transformations and statistical analyses were performed using SAS© version 9.4.

## Results

Of the 6115 US patients identified with an amyloidosis claim, 302 had an additional qualifier for ATTRv amyloidosis. After applying further criteria, the final sample included 141 patients with newly diagnosed ATTRv amyloidosis and 423 matched controls (Table 1).

The mean (SD) age at diagnosis across patients in the study was 62.5 (14.2) years, with the majority (76%) of patients having a diagnosis at age 55 years or older; 53.9% were female.

Patients with ATTRv amyloidosis presented with considerable comorbidity burden prior to diagnosis, with a mean (SD) CCI of 2.7 (3.0) and 5.1 (2.7) chronic conditions compared to a CCI of 1.1 (1.9) and 3.2 (2.3) chronic conditions for matched controls (*p* < 0.001). Primary care providers were the most common physicians seen by patients with ATTRv amyloidosis in the year prior to diagnosis (46.1% vs. 54.4% for matched controls); followed by neurologists (4.3% vs. 0.5%), gastroenterologists (4.3% vs. 1.4%), cardiologist (3.5% vs. 2.4%), rheumatologist (3.5% vs. 1.4%), and dermatologists (2.8% vs. 3.8%) (*p* = 0.018; Table 2).

**Table 2:** Baseline demographics and comorbidities

	Newly diagnosed ATTRv Amyloidosis patients N = 141	Matched controls^a^ N = 423	*p* value
Age, year, mean (SD)	62.5 (14.3)	62.5 (14.2)	n/a^d^
18–34, n (%)	6 (4.3)	18 (4.3)	
35–54	27 (19.1)	81 (19.1)	
55–64	52 (36.9)	156 (36.9)	
65+	56 (39.7)	168 (39.7)	
Female, n (%)	76 (53.9)	228 (53.9)	n/a^d^
Region, n (%)			
Midwest	26 (18.4)	78 (18.4)	n/a^d^
Northeast	47 (33.3)	141 (33.3)	
South	55 (39.0)	165 (39.0)	
West	13 (9.2)	39 (9.2)	
Insurance type, n (%)^b^			0.087
PPO/POS	99 (70.2)	239 (56.5)	
HMO/EPO	8 (5.7)	30 (7.1)	
CDHP/HDHP	14 (9.9)	72 (17.0)	
Comprehensive	20 (14.2)	80 (18.9)	
Charlson comorbidity index, mean (SD)	2.7 (3.0)	1.1 (1.9)	< 0.001
Number of chronic conditions, mean (SD)	5.1 (2.7)	3.2 (2.3)	< 0.001
Health care provider, n (%)			0.018
Primary care	65 (46.1)	230 (54.4)	
Cardiologist	5 (3.5)	10 (2.4)	
Dermatologist	4 (2.8)	16 (3.8)	
Gastroenterologist	6 (4.3)	6 (1.4)	
Neurologist	6 (4.3)	2 (0.5)	
Rheumatologist	5 (3.5)	6 (1.4)	
Other^c^/unknown	50 (35.5)	153 (36.2)	

*ATTRv* hereditary transthyretin, *CDHP/HDHP* consumer directed health plan/high deductible health plan, *EPO* exclusive provider organization, *HMO* health maintenance organization, *PPO/POS* preferred provider organizations/point of service

^a^Matched with age, gender, and region

^b^Two matched controls had missing/unknown insurance type

^c^Includes podiatrists and individual specialties with count < 5

^d^Matched exactly

During the 5-year look-back period, the occurrence of comorbid conditions or symptoms was common among patients with ATTRv amyloidosis and more frequent relative to matched controls. Dyspnea was the most common condition, occurring in 49.6% of patients with ATTRv amyloidosis and 25.8% of matched controls (*p* < 0.001), followed by diabetes, nausea/vomiting, neuropathy, constipation, and congestive heart failure occurring in more than 20% of patients with ATTRv (Table 3). For patients with ATTRv amyloidosis, the first observed occurrence of nearly all comorbidities (except ocular conditions) was observed in each of the 5 look-back years. For certain conditions, such as neuropathy, congestive heart failure, hypertrophic cardiomyopathy, ventricular hypertrophy, constipation, weight loss, hypotension, and renal failure, the first observed occurrence appeared to be highest in the year prior to diagnosis, following lower, and sometimes, gradually increasing frequencies in the years prior. For matched controls, the first occurrence of the selected comorbidities appeared evenly distributed across the 5 look-back years without a rise in the most recent year (Table 3).

**Table 3:** Selected comorbidities during the 5 years prior to ATTRv amyloidosis diagnosis

	Newly diagnosed ATTRv amyloidosis patients N = 141	Matched controls^a^ N = 423	*P* value	Newly diagnosed ATTRv amyloidosis patients N = 141	Matched controls^a^ N = 423	*P* value	Newly diagnosed ATTRv amyloidosis patients N = 141	Matched controls^a^ N = 423	*P* value	Newly diagnosed ATTRv amyloidosis patients N = 141	Matched controls^a^ N = 423	*P* value
	Ocular, and severe organ dysfunction or failure											
	Glaucoma			Vitreous opacity			Renal failure			Organ transplant		
N (%)	21 (14.9)	53 (12.5)	0.471	12 (8.5)	18 (4.3)	0.051	28 (19.9)	24 (5.7)	< 0.001	4 (2.8)	0 (0.0)	< 0.001
First evidence occurred, n (%)			0.285			0.208			< 0.001			0.007
No evidence	120 (85.1)	370 (87.5)		129 (91.5)	405 (95.7)		113 (80.1)	399 (94.3)		137 (97.2)	423 (100.0)	
Pre Y1	1 (0.7)	0 (0)		2 (1.4)	3 (0.7)		13 (9.2)	4 (0.9)		2 (1.4)	0 (0)	
Pre Y2	1 (0.7)	1 (0.2)		2 (1.4)	4 (0.9)		6 (4.3)	6 (1.4)		1 (0.7)	0 (0)	
Pre Y3	3 (2.1)	9 (2.1)		4 (2.8)	2 (0.5)		2 (1.4)	3 (0.7)		0 (0)	0 (0)	
Pre Y4	1 (0.7)	10 (2.4)		2 (1.4)	3 (0.7)		2 (1.4)	3 (0.7)		1 (0.7)	0 (0)	
Pre Y5	15 (10.6)	33 (7.8)		2 (1.4)	6 (1.4)		5 (3.5)	8 (1.9)		0 (0)	0 (0)	

	Gastrointestinal											
	Diarrhea			Constipation			Nausea/vomiting			Weight loss		
N (%)	25 (17.7)	47 (11.1)	0.041	35 (24.8)	49 (11.6)	< 0.001	38 (27.0)	55 (13.0)	< 0.001	19 (13.5)	30 (7.1)	0.020
First evidence occurred, n (%)			0.352			0.005			< 0.001			0.003
No evidence	116 (82.3)	376 (88.9)		106 (75.2)	374 (88.4)		103 (73.0)	368 (87.0)		122 (86.5)	393 (92.9)	
Pre Y1	5 (3.5)	8 (1.9)		11 (7.8)	11 (2.6)		6 (4.3)	5 (1.2)		10 (7.1)	5 (1.2)	
Pre Y2	4 (2.8)	11 (2.6)		9 (6.4)	14 (3.3)		4 (2.8)	15 (3.5)		0 (0)	7 (1.7)	
Pre Y3	6 (4.3)	11 (2.6)		5 (3.5)	5 (1.2)		8 (5.7)	17 (4.0)		3 (2.1)	6 (1.4)	
Pre Y4	5 (3.5)	6 (1.4)		5 (3.5)	11 (2.6)		9 (6.4)	8 (1.9)		2 (1.4)	6 (1.4)	
Pre Y5	5 (3.5)	11 (2.6)		5 (3.5)	8 (1.9)		11 (7.8)	10 (2.4)		4 (2.8)	6 (1.4)	

	Musculoskeletal and cardiovascular											
	Spinal stenosis			Carpal tunnel			Congestive heart failure			Hypotension		
N (%)	23 (16.3)	43 (10.2)	0.049	13 (9.2)	28 (6.6)	0.030	33 (23.4)	25 (5.9)	< 0.001	26 (18.4)	26 (6.1)	< 0.001
First evidence occurred, n (%)			0.012			0.104			< 0.001			< 0.001
No evidence	118 (83.7)	380 (89.8)		128 (90.8)	395 (93.4)		108 (76.6)	398 (94.1)		115 (81.6)	397 (93.9)	
Pre Y1	3 (2.1)	16 (3.8)		4 (2.8)	4 (0.9)		11 (7.8)	6 (1.4)		11 (7.8)	6 (1.4)	
Pre Y2	5 (3.5)	6 (1.4)		2 (1.4)	6 (1.4)		6 (4.3)	3 (0.7)		5 (3.5)	6 (1.4)	
Pre Y3	3 (2.1)	6 (1.4)		3 (2.1)	2 (0.5)		7 (5.0)	3 (0.7)		3 (2.1)	5 (1.2)	
Pre Y4	2 (1.4)	8 (1.9)		4 (2.8)	8 (1.9)		6 (4.3)	4 (0.9)		2 (1.4)	4 (0.9)	
Pre Y5	10 (7.1)	7 (1.7)		0 (0)	8 (1.9)		3 (2.1)	9 (2.1)		5 (3.5)	5 (1.2)	

	Cardiovascular (continued)											
	Dyspnea			Ventricular hypertrophy			Hypertrophic cardiomyopathy			Restrictive cardiomyopathy		
N (%)	70 (49.6)	109 (25.8)	< 0.001	28 (19.9)	24 (5.7)	< 0.001	8 (5.7)	2 (0.5)	< 0.001	11 (7.8)	8 (1.9)	< 0.001
First evidence occurred, n (%)			< 0.001			< 0.001			0.003			< 0.001
No evidence	71 (50.4)	314 (74.2)		113 (80.1)	399 (94.3)		133 (94.3)	421 (99.5)		130 (92.2)	415 (98.1)	
Pre Y1	9 (6.4)	15 (3.5)		8 (5.7)	6 (1.4)		3 (2.1)	1 (0.2)		1 (0.7)	0 (0)	
Pre Y2	12 (8.5)	15 (3.5)		6 (4.3)	6 (1.4)		2 (1.4)	0 (0)		4 (2.8)	0 (0)	
Pre Y3	9 (6.4)	21 (5.0)		5 (3.5)	6 (1.4)		1 (0.7)	1 (0.2)		1 (0.7)	2 (0.5)	
Pre Y4	15 (10.6)	16 (3.8)		4 (2.8)	1 (0.2)		1 (0.7)	0 (0)		3 (2.1)	0 (0)	
Pre Y5	25 (17.7)	42 (9.9)		5 (3.5)	5 (1.2)		1 (0.7)	0 (0)		2 (1.4)	6 (1.4)	

	Nervous system and metabolic								
	Neuropathy			Incontinence			Diabetes		
N (%)	37 (26.2)	25 (5.9)	< 0.001	20 (14.2)	23 (5.4)	< 0.001	56 (39.7)	107 (25.3)	0.001
First evidence occurred, n (%)			< 0.001			0.005			0.022
No evidence	104 (73.8)	398 (94.1)		121 (85.8)	400 (94.6)		85 (60.3)	316 (74.7)	
Pre Y1	17 (12.1)	7 (1.7)		2 (1.4)	2 (0.5)		4 (2.8)	8 (1.9)	
Pre Y2	7 (5.0)	4 (0.9)		1 (0.7)	4 (0.9)		6 (4.3)	12 (2.8)	
Pre Y3	6 (4.3)	4 (0.9)		5 (3.5)	2 (0.5)		4 (2.8)	3 (0.7)	
Pre Y4	1 (0.7)	3 (0.7)		6 (4.3)	7 (1.7)		10 (7.1)	16 (3.8)	
Pre Y5	6 (4.3)	7 (1.7)		6 (4.3)	8 (1.9)		32 (22.7)	68 (16.1)	

*ATTRv* hereditary transthyretin

^a^Matched with age, gender, and region

The occurrence of the selected diagnostic testing was also more frequent among patients with ATTRv amyloidosis versus matched controls in the 5 years leading up to diagnosis. Echocardiogram (56.7% vs. 27.0%; *p* < 0.001), followed by tissue biopsy or genetic testing (34.8% vs. 20.8%; *p* < 0.001) and blood/urine testing (34.8% vs. 9.5%; *p* < 0.001), was the most common in both groups, while cardiac magnetic resonance imaging (7.1% vs. 0.2%; *p* < 0.001) and pyrophosphate imaging (2.1% vs. 0.0%; *p* = 0.003) rates were low in both groups (Table 4).

**Table 4:** Diagnostic testing during the 5 years prior to ATTRv amyloidosis diagnosis

	Newly diagnosed ATTRv Amyloidosis Patients N = 141	Matched controls^a^ N = 423	*p* value	Newly diagnosed ATTRv Amyloidosis Patients N = 141	Matched controls^a^ N = 423	*p* value	Newly diagnosed ATTRv Amyloidosis Patients N = 141	Matched controls^a^ N = 423	*p* value
	Pyrophosphate imaging (PYP)			Cardiac magnetic resonance imaging (MRI)			Echocardiogram		
N (%)	3 (2.1)	0 (0.0)	0.003	10 (7.1)	1 (0.2)	< 0.001	80 (56.7)	114 (27.0)	< 0.001
First evidence occurred	138 (97.9)	423 (100.0)	0.003	131 (92.9)	422 (99.8)	< 0.001	61 (43.3)	309 (73.0)	< 0.001
No evidence									
Pre Y1	3 (2.1)	0 (0)		6 (4.3)	1 (0.2)		12 (8.5)	19 (4.5)	
Pre Y2	0 (0)	0 (0)		1 (0.7)	0 (0)		11 (7.8)	14 (3.3)	
Pre Y3	0 (0)	0 (0)		1 (0.7)	0 (0)		11 (7.8)	19 (4.5)	
Pre Y4	0 (0)	0 (0)		2 (1.4)	0 (0)		19 (13.5)	21 (5.0)	
Pre Y5	0 (0)	0 (0)		0 (0)	0 (0)		27 (19.1)	41 (9.7)	

	Blood/urine testing		*p* value	Tissue biopsy or genetic testing^b^		*p* value			
N (%)	49 (34.8)	40 (9.5)	< 0.001	49 (34.8)	88 (20.8)	< 0.001			
First evidence occurred			< 0.001			0.003			
No evidence	92 (65.2)	383 (90.5)		92 (65.2)	335 (79.2)				
Pre Y1	14 (9.9)	13 (3.1)		11 (7.8)	14 (3.3)				
Pre Y2	7 (5.0)	6 (1.4)		7 (5.0)	12 (2.8)				
Pre Y3	10 (7.1)	7 (1.7)		13 (9.2)	14 (3.3)				
Pre Y4	10 (7.1)	7 (1.7)		10 (7.1)	21 (5.0)				
Pre Y5	8 (5.7)	7 (1.7)		8 (5.7)	27 (6.4)				

*ATTRv* Hereditary transthyretin

^a^Matched with age, gender, and region

^b^Tissue biopsy tests limited to peripheral, cardiac, salivary, rectal, fat pad areas of the body and nerves, and genetic tests include those used to analyze nucleic acid for abnormalities that may be indicative of a variety of disorders

The occurrence of ED visits and hospitalization was also much more common among patients with ATTRv amyloidosis relative to controls. Nearly half (47.5%) of patients with ATTRv amyloidosis had a hospitalization in the 5 years prior to diagnosis (matched controls, 24.3%); and 60.3% of patients with ATTRv amyloidosis had an ED visit in the 5 years before diagnosis versus 47.0% of matched controls (results not shown in table). For patients with ATTRv amyloidosis, ED visits and hospitalizations (first or any occurrence) increased steadily in each look-back year until diagnosis (from 11.3 to 21.3% hospitalizations; from 26.2 to 34.8% ED visits) (results not shown in table).

**Fig. 2:**
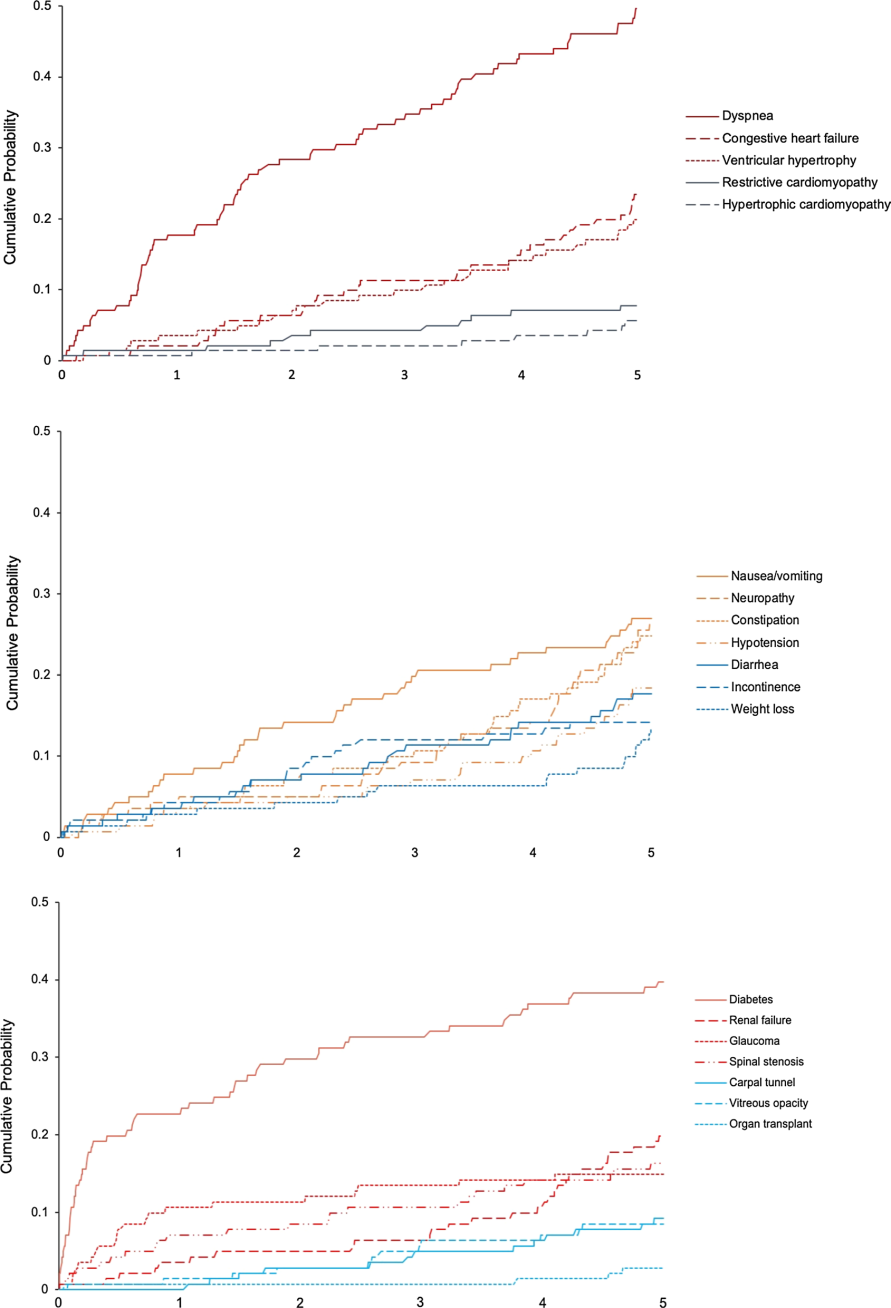
First observed evidence of comorbidities during the 5 years prior to diagnosis

**Fig. 3:**
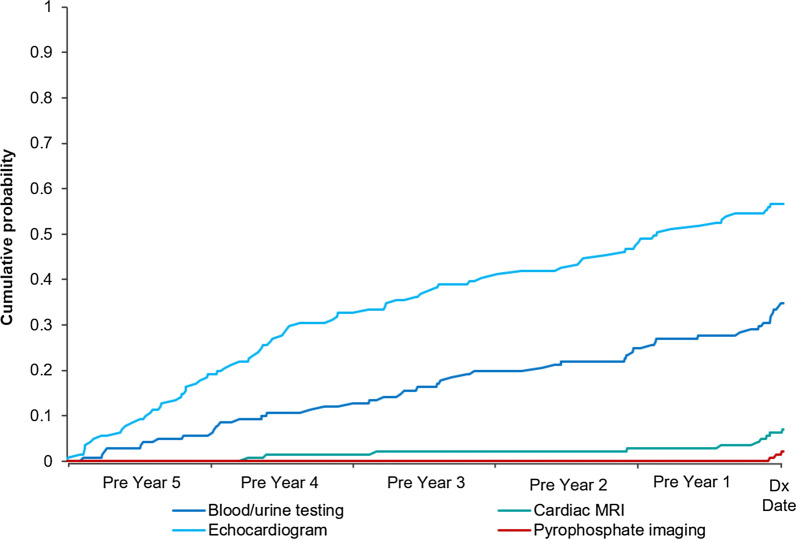
First observed evidence of diagnostic testing during the 5 years prior to diagnosis. *MRI* magnetic resonance imaging

Cumulative probability plots (Figs. 2, 3) show consistent results for patients with ATTRv amyloidosis with the first occurrence of comorbidities and selected diagnostic testing often being early in the 5-year look-back period prior to diagnosis.

## Discussion

In this retrospective analysis using US commercial and Medicare supplemental insurance claims data, we found that patients with ATTRv amyloidosis experienced significant comorbidity burden and healthcare utilization in the 5 years prior to diagnosis. All conditions and symptoms were higher among patients with ATTRv amyloidosis compared to matched controls without the disease, suggesting that comorbidities may be associated with the condition. This difference was observed in each of the 5 look-back years prior to diagnosis, suggesting manifestation of ATTRv amyloidosis may go back several years before a diagnosis is made.

In addition, selected diagnostic testing and acute care (i.e., ED visits, hospitalizations) were also more common in the 5 years prior to diagnosis for patients with ATTRv amyloidosis compared to controls. Utilization of cardiac imaging, biopsy or genetic testing, and blood/urine testing was higher in each of the look-back years relative to controls. The proportions of patients with ED visits and hospitalization during the 5-year look-back period were significantly higher among those with ATTRv amyloidosis compared to controls, with hospitalization nearly doubled.

This study provides evidence of the clinical manifestation of ATTRv amyloidosis and associated healthcare utilization up to several years before a diagnosis of ATTRv in the US. The early presence of these potential markers of disease may point to delays in the diagnosis of the condition. In addition, the presence of neuropathic and cardiac symptoms may suggest the development of polyneuropathy and cardiomyopathy, respectively, which are serious manifestations of the disease. The identification and recognition of common markers of early disease could prompt patients’ screening and, in turn, accelerate early diagnosis and treatment.

This real-world study adds to the limited literature on the ATTRv amyloidosis patient journey through examining the clinical characteristics and healthcare utilization in the years leading up to a diagnosis of ATTRv amyloidosis. McCausland et al. [27] examined the diagnostic journey for patients with AL amyloidosis using interview and survey data. While this is a different type of amyloidosis, the authors found that diagnostic delay was common due to variability in initial symptom manifestations, as with ATTRv amyloidosis, leading to potential misdiagnoses [27]. In a retrospective chart review, Bishop et al. [24] found that certain factors, including having ATTR amyloidosis, predicted delayed diagnosis of cardiac amyloidosis. They also reported that it is not uncommon for patients to experience long-standing cardiac symptoms with without an amyloidosis diagnosis, and that a delay of more than 2 years resulted in increased disease burden and increased evidence of myocardial injury and failure [24].

In addition, we observed that the occurrence of most clinical manifestations appeared to be highest in the year prior to diagnosis. The rise in occurrence of ED visits and hospitalizations with each year over the 5-year look-back period, suggests an increased need for acute care prior to diagnosis of ATTRv.

### Limitations

This study had several limitations. First, our approach to identifying patients with ATTRv amyloidosis has not been validated using medical records; however, the majority (92%) of patients were identified with qualifying diagnoses codes for hereditary amyloidosis (E85.1 or E85.2), increasing our confidence that the correct population was identified. The 15-day requirement for diflunisal use was chosen to exclude short-term pain use; and while the use of liver transplant could have captured patients without the disease, only six patients were included in the analysis based on this qualifier (Table 1). Second, as this study examined data during the 5 years prior to diagnosis, we may have misclassified existing comorbidities or complications as being first observed during the 5-year look-back period. Third, miscoding in the claims data could have led to an inaccurate estimation of the frequency of comorbidities, complications, and health-services utilization. For example, the prevalence of carpal tunnel in the ATTRv amyloidosis cohort was lower than that reported in previous literature [17, 24]. Fourth, while mutation type is known to impact clinical manifestations (e.g., neurologic, cardiac, GI), the potential effect of genotype on the frequency of comorbidities, complications and services utilization could not be examined in this study as genotype information is not available in the source claims data. Lastly, results may not be generalizable to patients without continuous enrollment in a healthcare plan, including those without commercial coverage.

## Conclusion

Patients with ATTRv amyloidosis experience multiple neurological, cardiovascular, and other clinical manifestations, testing, and hospitalization prior to diagnosis. Moreover, many patients with ATTRv amyloidosis appear to have manifestations of the disease several months or years before diagnosis, pointing to opportunities for earlier identification. Improved understanding of the patient journey before a diagnosis of ATTRv amyloidosis may accelerate clinical recognition of the disease and lead to early diagnosis.

## Data Availability

The datasets generated during and/or analyzed during the current study are not publicly available as the MarketScan data were used under license for the current study, but are available from the authors upon reasonable request and with permission of IBM.

## Footnotes

1 MarketScan is a trademark of IBM Corporation in the United States and other countries.
